# Changes of the absorption cross section of Si nanocrystals with temperature and distance

**DOI:** 10.3762/bjnano.8.231

**Published:** 2017-11-06

**Authors:** Michael Greben, Petro Khoroshyy, Sebastian Gutsch, Daniel Hiller, Margit Zacharias, Jan Valenta

**Affiliations:** 1 Department of Chemical Physics and Optics, Faculty of Mathematics and Physics, Charles University, Ke Karlovu 3, 121 16 Prague 2, Czech Republic; 2 Institute of Organic Chemistry and Biochemistry, Czech Academy of Sciences, Flemingovo namesti 2, 160 00 Prague 6, Czech Republic; 3 Faculty of Engineering, IMTEK, Albert-Ludwigs-University Freiburg, Georges-Köhler-Allee 103, 79110 Freiburg, Germany

**Keywords:** absorption cross section, average lifetime, nanocrystal distance, photoluminescence decay, silicon nanocrystals

## Abstract

The absorption cross section (ACS) of silicon nanocrystals (Si NCs) in single-layer and multilayer structures with variable thickness of oxide barriers is determined via a photoluminescence (PL) modulation technique that is based on the analysis of excitation intensity-dependent PL kinetics under modulated pumping. We clearly demonstrate that roughly doubling the barrier thickness (from ca. 1 to 2.2 nm) induces a decrease of the ACS by a factor of 1.5. An optimum separation barrier thickness of ca. 1.6 nm is calculated to maximize the PL intensity yield. This large variation of ACS values with barrier thickness is attributed to a modulation of either defect population states or of the efficiency of energy transfer between confined NC layers. An exponential decrease of the ACS with decreasing temperature down to 120 K can be explained by smaller occupation number of phonons and expansion of the band gap of Si NCs at low temperatures. This study clearly shows that the ACS of Si NCs cannot be considered as independent on experimental conditions and sample parameters.

## Introduction

For decades, silicon – an abundant, nontoxic material with high attainable purity – has been a dominant material for microelectronics and photovoltaics. However, the constantly increasing energy consumption and environmental issues challenge researchers to develop fundamentally new concepts to overcome the limitations of current technologies. The nanocrystalline form of silicon, which reveals all advantages of the quantum confinement effect [1], is a promising candidate for the development of a new generation of Si photovoltaic and photonic devices [2]. SiO_2_-embedded silicon nanocrystals (Si NCs) can be relatively easy integrated into current CMOS technology. In photovoltaics, nanocrystalline Si is a promising material for the top cell of all-Si tandem cells that can theoretically reach efficiencies much above the Shockley–Queisser limit of 31% for single-junction solar cells [2]. Current injection into Si NCs can be utilized in Si-based light emitting diodes or displays [3]. A device fabrication process demands an effective control of size, shape and density of Si NCs. All those requirements can be met via the superlattice approach in combination [4] with the phase-separation of sub-stoichiometric oxides (SiO*_x_*) where the NC spacing in all three dimensions can be controlled.

The emission properties such as quantum yield (QY) of such Si NC/SiO_2_ multilayer (ML) structures were studied as a function of inter-nanocrystal distance, temperature, excitation and emission wavelength [5–6]. However, there is still little knowledge about one of the most crucial optical parameters for spectroscopic studies and the design of Si NC optoelectronic devices, which is related to the strength of light absorption: the absorption cross section (ACS), σ. The ACS directly reflects the probability of optical transitions and is defined as the ratio [7] between photon absorption rate for a single NC and the photon flux, which in fact provides a relationship [8] between the NC concentration and the optical density of the sample. Consequently, if the absolute value of the ACS is determined, the concentration of NCs in a studied sample can be directly calculated from measurements of the optical absorption coefficient [9]. The NC concentration is necessary for many scientific studies and practical applications such as biolabeling [3]. Besides this, the ACS is related to the transition oscillator strength and therefore, is a very useful parameter for a variety of theoretical calculations as it defines an upper limit of the exciton radiative lifetime of a NC [8]. Though the ACS is a very important quantity for practice it is not easily accessible experimentally, which explains a very limited number of reports in the literature. Recently we presented a comparative study of ACS determination by two completely independent methods including a photoluminescence (PL) modulation technique [10]. In this work, we employ this procedure to analyze the dependence of the ACS of ML structures on mainly two important parameters: inter-nanocrystal distance and temperature. It will be shown that, contrary to popular belief, the ACS depends on the temperature. Moreover, we combine our knowledge on QY and ACS to derive the optimum separation barrier thickness to maximize PL intensity yield at a given excitation intensity.

## Experimental

The investigated ML samples were deposited as alternating layers of silicon-rich silicon oxynitride (SRON: SiO*_x_*N*_y_*) with 4.5 nm thickness and of stoichiometric SiO_2_ (1, 1.6, 2.2 or 2.8 nm thick) on fused silica substrates by plasma-enhanced chemical vapor deposition (PECVD). On top and below the superlattice stack, 10 nm of SiO_2_ were deposited as a buffer and capping layer, respectively. The samples were subsequently annealed in a quartz tube furnace at 1150 °C for 1 h in high-purity N_2_ in order to form Si NCs and then passivated by annealing in H_2_ at 500 °C for defect passivation. In addition to ML samples, one single layer (SL) sample with a thick SRON 200 nm monolayer without barriers was taken for comparison. The SRON stoichiometry parameter was almost constant y = 0.23 ± 0.002 in all samples while the *x* value was chosen as 0.93 and 1.1 for ML and SL samples, respectively. For further details on the sample preparation as well as structural properties of the samples see our recent paper [11].

The PL experiments were performed under excitation with a 405 nm diode laser the beam of which was modulated using a quartz acousto-optic cell. The edge switching time is about 100 ns. The laser is coupled to a custom-made micro-spectroscopy set-up with an inverted microscope in the epifluorescence configuration with two detection branches for visible and near-infrared spectral regions, each one composed of an imaging spectrometer, and a camera for spectral and a photomultiplier for time-resolved PL detection. The output of the photomultipliers is coupled in a multichannel counting card (Becker-Hickl, MSA-300). The details on the set-up can be found in our recent paper [12]. Advantage of the micro-PL set-up is a good control of the excitation spot size and the selected detection area, which enable a quite precise determination of the excitation photon flux. For the low-temperature experiments the samples are placed in a cryostat (Janis ST-500).

## Results and Discussion

### ACS model

Let us consider the model originally presented by Kovalev et al. [13–14] and then slightly modified in our recent paper [10]. A Si NC is considered as a quasi-two-level system with only three possible NC occupation states: ground state, single (one e–h pair in a NC) and double (two e–h pairs in a NC) excited state. Assuming the corresponding occupations as *N*_0_, *N*_1_ and *N*_2_ we can obtain the system of three differential equations describing optical dynamics of above mentioned model:

[1]
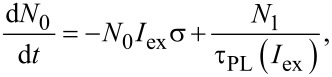


[2]
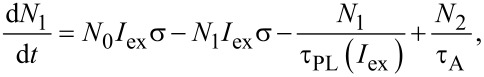


[3]
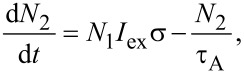


where σ describes the cross section for the absorption of photons, *I*_ex_ represents the excitation intensity expressed in areal photon flux (photons per second per square centimeter), τ_PL_(*I*_ex_) and τ_A_ stand for PL decay and Auger lifetime, respectively. Here the PL lifetime τ_PL_(*I*_ex_) is considered as a function of the excitation intensity (discussed below). The total population of luminescing nanocrystals is *N*_T_ = *N*_0_ + *N*_1_ + *N*_2_. To complete the model we should summarize all of its assumptions: (1) The Auger lifetime is considered to be power-independent and much shorter (for Si NCs in the nanosecond-range or shorter [15]) than τ_PL_(*I*_ex_). Therefore, all higher excited states are not taken into account supposing that Auger recombination efficiently quenches the population of double excited NCs. (2) The ground state and single-excited state roughly have the same ACS, σ, as we assume that the presence of one e–h pair does not influence the absorption of the second one (because of the relatively high [1] density of optical states (DOS) in Si NCs).

According to the first assumption, 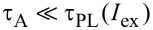 and therefore relaxation of biexcitons in Equation 3 for a given fraction of excitons *N*_1_ (Equation 2) can be considered as instantaneous on the slow time-scale evolution of *N*_1_. Consequently, from Equation 3 we assume the population *N*_2_ = *N*_1_*I*_ex_στ_A_ and Equation 2 can be rewritten as:

[4]
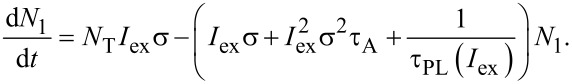


By taking into account [1] that the PL intensity is *I*_PL_ = *N*_1_/τ_r_ we can write the solution of Equation 4 in the form:

[5]
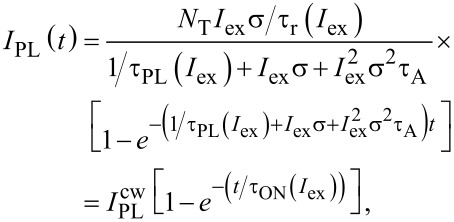


where τ_r_ stands for the radiative lifetime, which is believed to be independent on the excitation power, and τ_ON_ is the onset (ON) lifetime.

Equation 5 represents the most general solution of Equations 1–3. Consequently, from Equation 5 we derive:

[6]
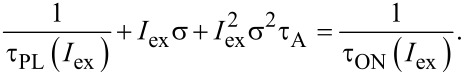


The solution of Equation 6 defines the ACS as a function of τ_ON_(*I*_ex_), τ_PL_(*I*_ex_) and even τ_A_:

[7]
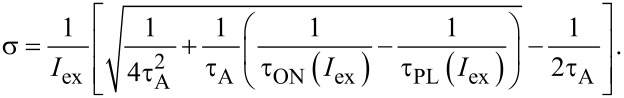


However, the Auger decay time τ_A_ is not easily determined (literature reports values within a broad range from picoseconds [15] to nanoseconds [16]). Therefore, we have to avoid strong-pumping regimes where double-excitation of NCs takes place.

Assuming *N*_2_/*N*_1_→0 we have *I*_ex_στ_A_→0 (see Equation 3) and the Auger part *I*_ex_σ(*I*_ex_στ_A_) is negligibly small in comparison with the term *I*_ex_σ in Equation 5 and can be neglected in regimes of low and moderate excitation powers:

[8]
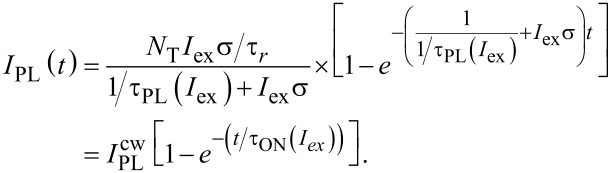


Thus, Equation 6 simplifies to the well-known equation [10,17–18] that will be used throughout for the ACS determination in this paper:

[9]
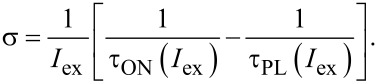


### Average lifetime calculations

**Figure 1:**
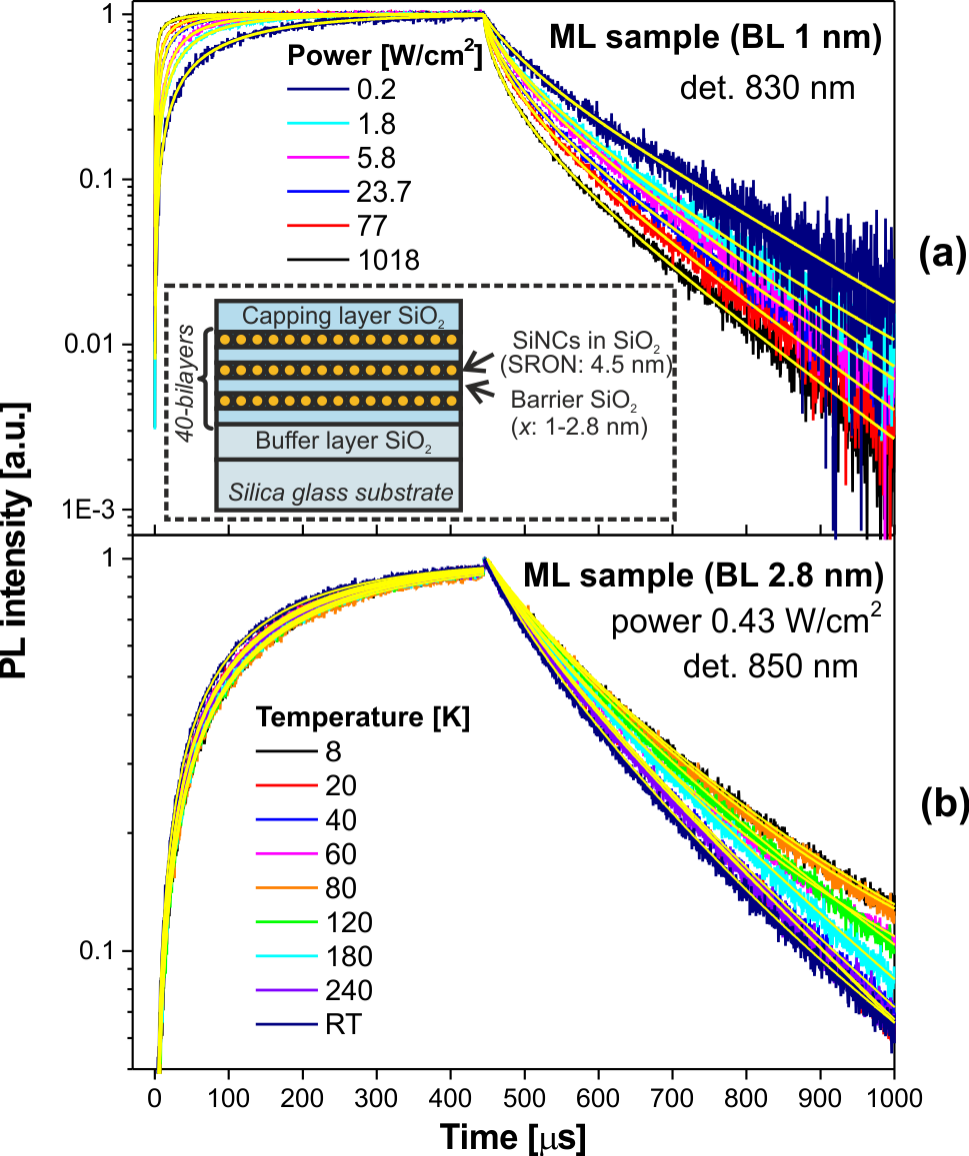
Time-resolved PL signals of multilayer samples (barrier layer (BL) is 1 nm (a) or 2.8 nm (b)) detected at 830 nm (a) and 850 nm (b) and excited by square pulses, as well as their corresponding fits by Equations 10–13 as a function of a) power density ranging from 0.2 to 1018 W/cm^2^ at room temperature and b) temperature ranging from 8 K up to room temperature. The inset illustrates the multilayer sample structure.

Though very often PL transients of Si NCs are well fitted by stretched exponential function [1], there is a number of reports where stretched exponential fit fails for both colloidal [19–20] and matrix-embedded NCs [21–22]. Instead, sometimes a log-normal rate distribution model [23] could be helpful to describe the de-excitation dynamics of the NC ensemble. Unfortunately, neither stretched exponential nor log-normal decay models can fit our experimentally measured PL curves (Figure 1).

Our goal is to extract the average ON and PL lifetimes as a function of different parameters. Thus, two fitting models were employed for this and the resulting lifetimes were compared. In the first approach, the sum of *N* mono-exponentials (ME) was utilized:

[10]
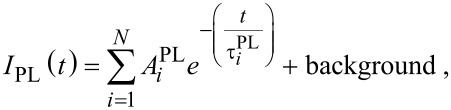


where *A_i_* and τ*_i_* are the according amplitudes and lifetime parameters, respectively; the background is a “constant” signal background level of a detector.

In the second approach, a combination of one mono- and one stretched-exponential (MSE) was used:

[11]
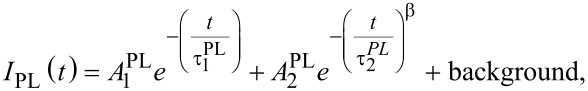


where β is the dispersion factor, which varies from 0 to 1.

Usually the onset (rise) PL dynamics is not analyzed in processing of time-resolved PL data. Recently we demonstrated that special attention needs to be paid to the excitation pulse length [24]. Here we show how to utilize the knowledge of the average ON lifetime. According to Equation 8 the PL onset kinetics for the two fitting models are described as:

[12]
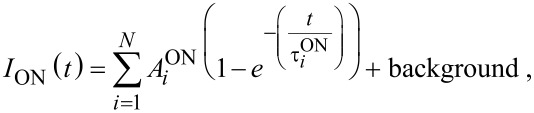


[13]
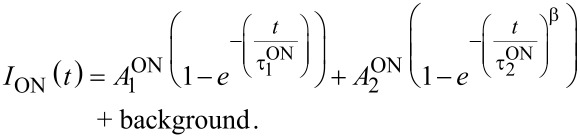


The average PL decay time of photons can be generally calculated [1] according to the statistical formula

[14]
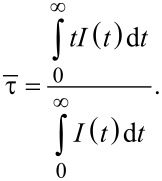


Finally, both average ON and PL lifetimes can be calculated by introducing Equation 10 and Equation 11 into Equation 14:

[15]
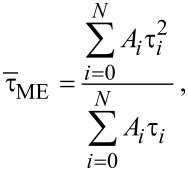


[16]
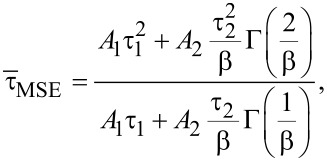


where Γ corresponds to the gamma function.

Although in this paper a precise data treatment was carried out we remind the reader about possible approximate calculations of average lifetimes for such complex decay kinetics [10,21].

### PL modulation technique

**Figure 2:**
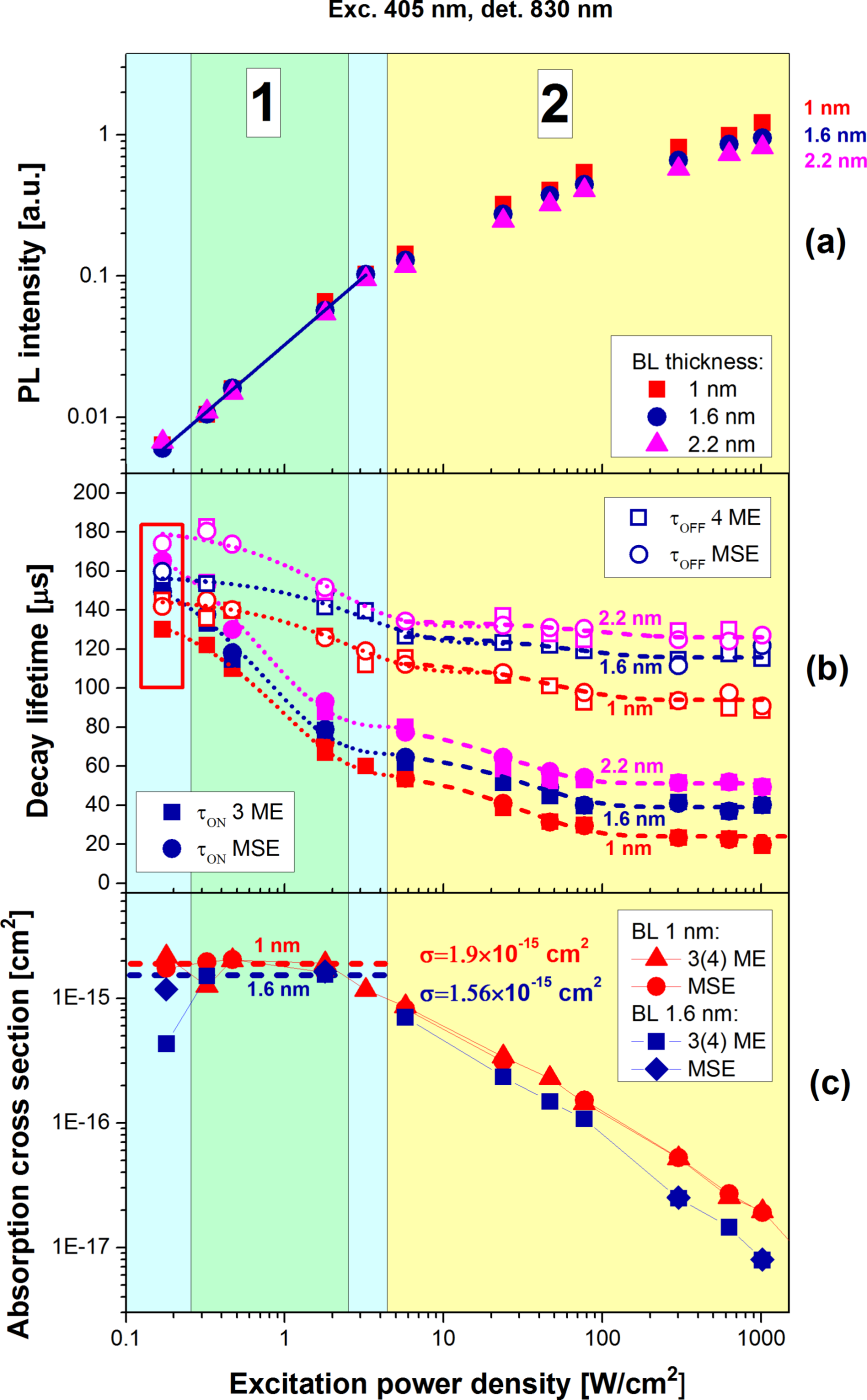
a) PL intensity under continuous-wave excitation as a function of the excitation photon flux for samples with 1 nm (squares), 1.6 nm (circles) and 2.2 nm (triangles) oxide barrier thickness. b) The ON (filled symbols) and PL (opened symbols) characteristic lifetimes extracted by fitting with either a combination of 3–4 mono-exponentials (ME) (squares) given by Equation 10 and Equation 12, or mono- and stretched exponentials (circles) (MSE) given by Equation 11 and Equation 13 and their corresponding exponential fits (dotted and dashed lines). c) The final ACS obtained from Equation 9 for samples with separation barriers of 1 and 1.6 nm. Throughout the figure the red, royal blue and pink colors represent samples with 1, 1.6 and 2.2 nm barrier thickness, respectively. The cyan and yellow areas stand for the linear regime and the saturation regime, respectively. The green area restricts the region of trusted ACS determination.

Spectrally resolved PL traces were measured at different temperatures while the excitation power was varied over four orders of magnitude (Figure 1). The power dependence of PL amplitudes (detected at 830 nm) from samples with different thicknesses of the oxide barrier layers (BL) are depicted in Figure 2a. The steady-state PL intensity in the low-excitation region 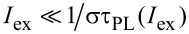 follows from Equation 8 and reveals a linear dependence on the power (Figure 2a):

[17]
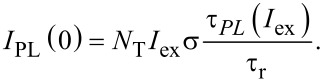


For moderate and high pump fluxes this intensity begins to saturate (Equation 8 and Equation 5, accordingly). Although the Auger-related PL saturation phenomena was utilized in several papers [14,25–26] for ACS determination, here we are forced to reject this approach as the saturation models cannot fit well our experimental data. Instead, we turn to the PL modulation technique that was described in detail recently [10] and exploit only the linear PL regimes.

The power dependence of both ON and PL lifetimes extracted by the ME and MSE methods is presented in Figure 2b. Both methods result in almost identical lifetime values. This indicates an independence of the average lifetime on fitting models, and each model describes the PL decay curves quite well. As expected [10], an increase of excitation power results in shortening of both ON and PL lifetimes (Figure 2b). Also the PL kinetics become more non-exponential, i.e., the distribution of lifetimes becomes broader [27] (Figure 1a). Thus, both characteristic lifetimes τ_ON_ and τ_PL_ are roughly equal at low excitation in agreement with Equation 9 while for higher pumping both lifetimes decrease exponentially with increasing pumping power. It is important to note a change of the slope of the lifetime decrease near the PL saturation level (which is usually ca. 1 W/cm^2^ for Si NCs) after the power was increased by approximately two orders of magnitude (Figure 2b). This transition gives evidence for the appearance of an additional mode (Auger recombination) in the decay process for pumping above the saturation threshold. To the best of our knowledge this has not been reported before for lifetimes. Moreover, τ_ON_ expectedly decreases faster than τ_PL_ with increase of excitation pumping as it is predicted by Equation 9.

Finally, the ACS was calculated by using Equation 9 and its variation with power is presented in Figure 2c. Normally, the ACS is considered [14] as a product of both DOS and the transition oscillator strength, neither of which is expected to be power-dependent. However, from Figure 2c it follows that the ACS is gradually decreasing when pumping near and above the saturation. Firstly, we have to remind that Equation 9 is valid only under the assumption that the fraction of NCs with two e–h pairs is negligibly small (*N*_2_ ≈ 0). Otherwise, Equation 7 must be used instead. Above we restricted the validity of the described model to the linear power regime and therefore, excitation powers above saturation will not be considered. Secondly, both ON and PL lifetimes are almost equal at very low excitation and, therefore, the ACS is noisy. In between, there is a very narrow intermediate region of excitation powers where the described model is valid and the ACS can be reliably determined [10] as a constant value (Figure 2, green region).

**Figure 3:**
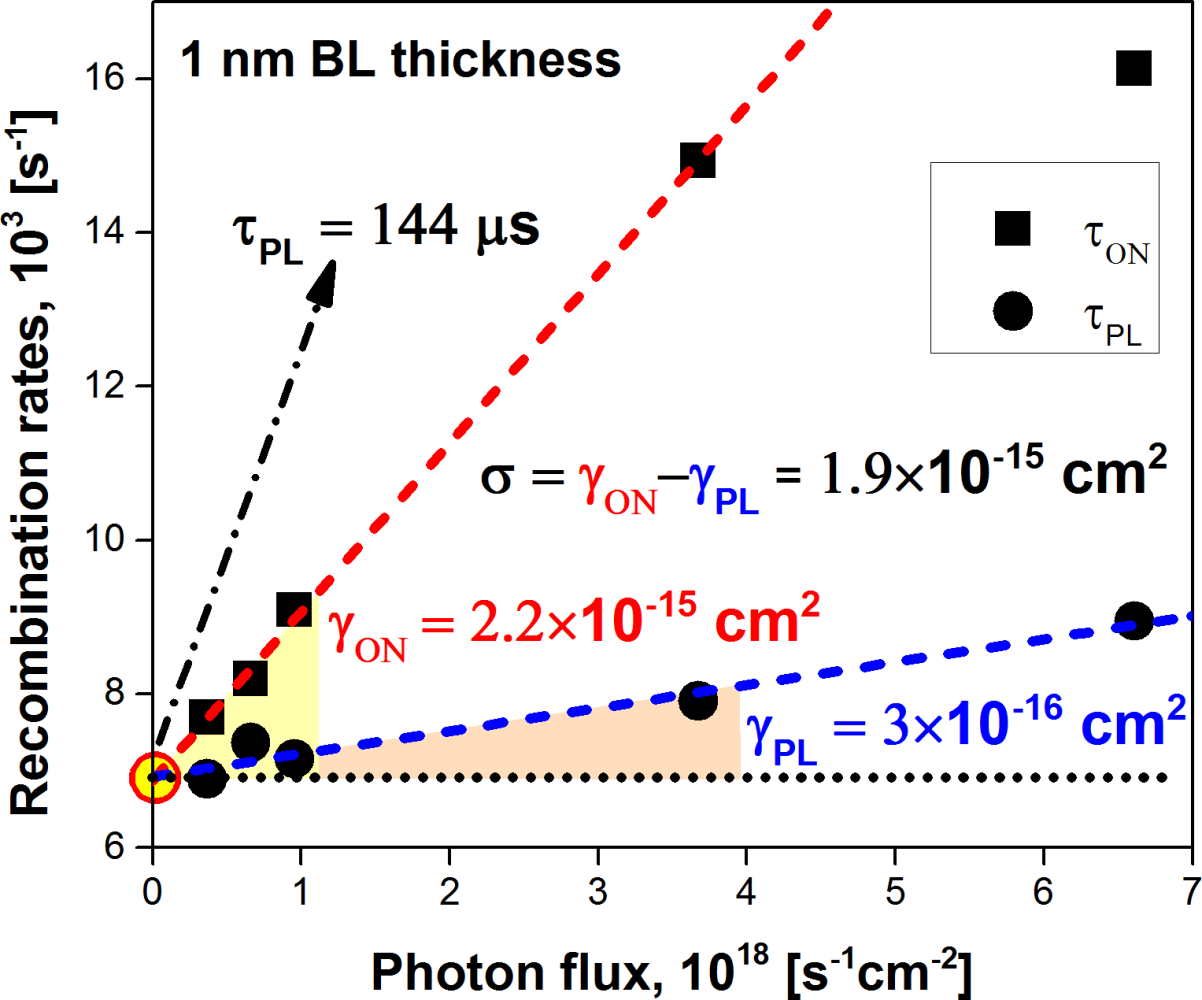
The extracted recombination rates γ_ON_ = 1/τ_ON_ and γ_PL_ = 1/τ_PL_ at *λ*_det_ = 830 nm of the ML sample with 1 nm BL thickness as functions of the excitation photon flux. The dashed lines are the corresponding linear function fits. The ACS is determined according to Equation 19.

As it follows from Equation 9, the inverse onset lifetime (onset rate), 1/τ_ON_, is a linear function of the photon flux *I*_ex_ with an offset given by the inverse decay PL lifetime (PL rate), 1/τ_PL_. Therefore, the ACS is usually determined directly as the slope of the function 1/τ_ON_(*I*_ex_). However, our experiments show that the PL lifetime, τ_PL_(*I*_ex_), is also a function of the excitation power [28] (Figure 2b). Assuming the radiative relaxation, τ_r_, to be independent on the power, this can be understood by the saturation of non-radiative recombination decay paths resulting in an increase of non-radiative lifetime τ_nr_. Analogous to 1/τ_ON_(*I*_ex_), the dependence 1/τ_PL_(*I*_ex_) can also be approximated with a linear function (Figure 3):

[18]
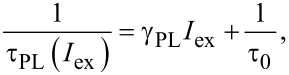


where the low-excitation lifetime is τ_0_ = τ_ON_(0) = τ_PL_(0), and γ_PL_ is the slope of the 1/τ_PL_(*I*_ex_) power dependence.

Finally, after substituting Equation 18 into Equation 9 we obtain

[19]
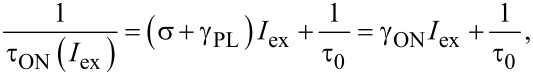


where γ_ON_ is the slope of the function 1/τ_ON_(*I*_ex_).

According to Equation 19, the correct ACS value must be calculated as the difference between the slopes of ON and PL rate σ = γ_ON_ − γ_PL_ (Figure 3) where both slopes are determined within the pumping range of linear dependence. Obviously, the variation of τ_PL_(*I*_ex_) was automatically included in the calculations presented in Figure 2c.

**Figure 4:**
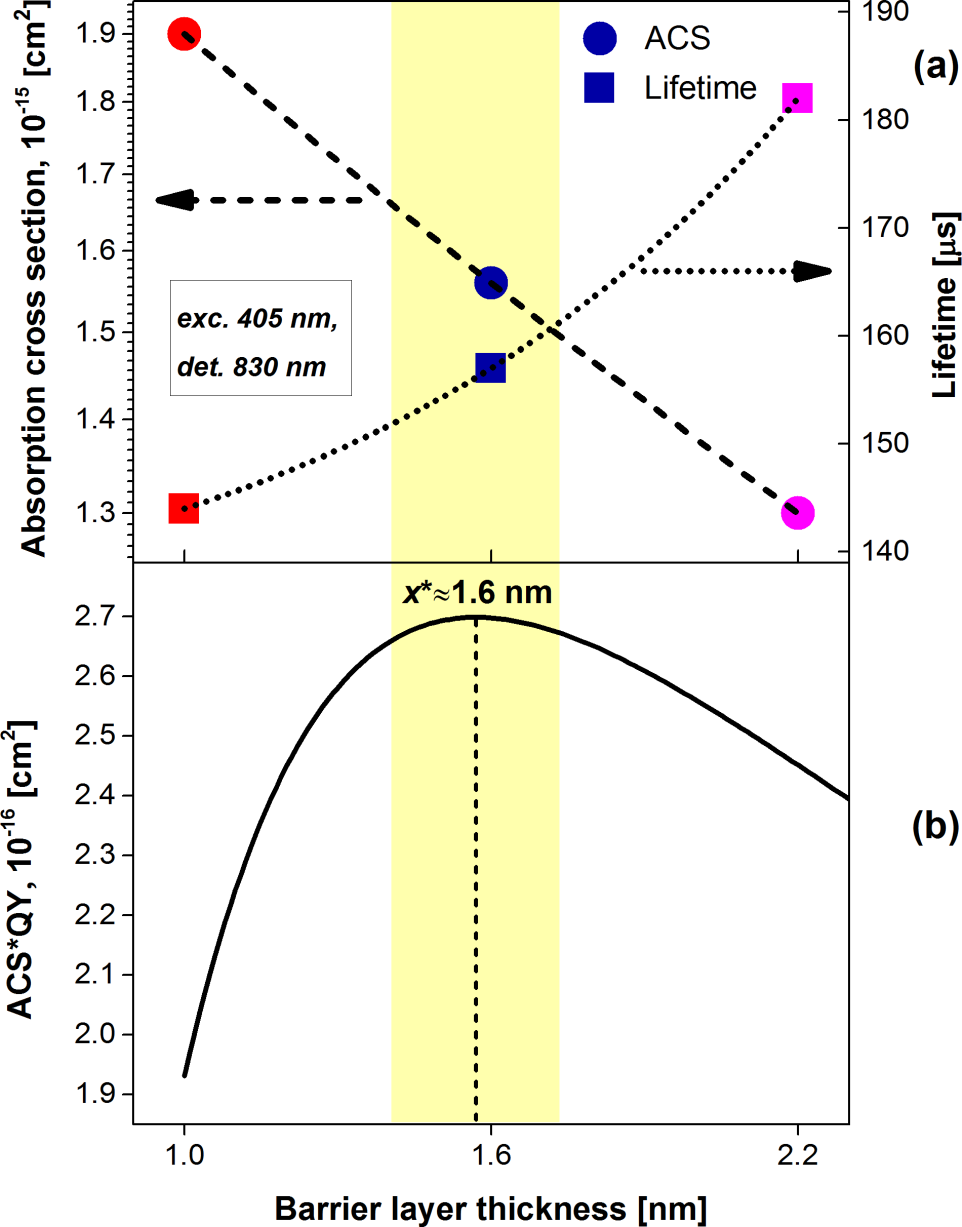
The dependence of (a) extracted PL lifetime τ(*x*) and calculated ACS σ(*x*) and (b) the product σ(*x*)·η(*x*) as a function of BL thickness *x*. The dashed and dotted lines in (a) are exponential fits. The corresponding fit parameters of the ACS according to Equation 20 are: *y*_0_ = 4.55·10^−16^ cm^2^, *A* = 1.445·10^−16^ cm^2^ and Δ*x* = 2.2366 nm.

In this way, we have calculated the ACS of ML samples in which the barrier thickness was varied (Figure 4a). As the separation increases, a transition from poorly separated NCs (1 nm or less) to a well-separated (above 2 nm) stack of confined layers is characterized [5] by an exponential increase of PL QY. In its turn, the ACS is decreasing with an increase of the NC separation barrier thickness (Figure 4a).

As presented in Figure 3**,** the PL modulation method directly gives us the true value of the lifetime τ_0_ (the low-excitation limit) as the intersection of ON–PL rate slopes (at *I*_ex_→0). In contrast to the ACS, the lifetime increases with an increase of the barrier thickness (Figure 4a). These variations of ACS and lifetime can be caused either by changes in the population of defect states or by changes of possible interactions between NC layers. On one hand, a lower number of defects for better separated NCs (which can be substantiated by higher QY [5]) can result in longer lifetimes, lower DOS and thus lower ACS (Figure 4a). On the other hand, in presence of exciton migration some neighboring NCs can work as antenna, because a NC can be excited either directly through photon absorption or indirectly through an energy transfer from a nearby NC. Therefore, an ACS enhancement is expected for the system of interacting NCs with thinner barrier. Moreover, as NC separation decreases, the hopping lifetime, τ_hop_, becomes shorter and, consequently, the PL lifetime decreases as well (Figure 4a). Separate experiments must be performed to figure out the real origin of the observed trend. Nevertheless, a similar (but much stronger) enhancement of ACS was reported by Priolo et al. [17] for a sample in which a substantial energy transfer was expected.

### An optimum inter-nanocrystal distance

Recently, we showed [5] that unlike ACS the PL QY is exponentially growing with increase of separation between confined NC layers. The quantum yield is a quantity that, in principle, must be independent on the number of absorbed photons [1]. In assumption that the internal quantum efficiency, η_I_ = τ_PL_/τ_r_, scales with the barrier thickness *x* in the same way as PL QY [5] (η = *N*_em_/*N*_abs_), i.e., the fraction of bright NCs *N*_em_ does not depend on *x*, the Equation 17 can be modified at the low-excitation limit as:

[21]
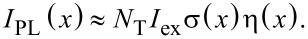


By using the dependence of σ(*x*) presented in Figure 4a and the previous results [5] we could approximate the ACS and the QY with exponential functions:

[20]
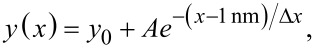


where y_0_ is an offset, *A* is an amplitude and Δ*x* is a characteristic distance.

For applications we usually aim to maximize the PL yield of photons at a certain *I*_ex_. Thus, the PL intensity *I*_PL_(*x*) is a product of a decreasing ACS and an increasing QY as the inter-NC distance becomes larger. An optimum barrier thickness of x* ≈ 1.6 nm can be easily found as the value that maximizes the function presented in Equation 21 (Figure 4b) for our ML structures.

### Temperature dependence of the ACS

Contrary to the common assumption, the ACS should be generally considered as temperature-dependent [5,29]. There are two mechanisms responsible for this dependence. First, for the phonon-assisted transitions the occupation number of phonons is an exponential function [1] of temperature containing the Bose–Einstein statistical factor:

[22]
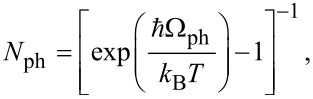


where Ω_ph_ is the typical (average) phonon energy and *k*_B_ is the Boltzmann constant.

Second, with varying temperatures the band gap of a NC is shifting. This changes the effective DOS at a certain energy. Following the phenomenological expression proposed by Cardona’s group [30] we can write:

[23]
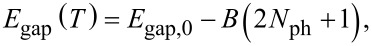


where *B* is a temperature-independent constant related to the strength of the electron–phonon interaction, and *E*_gap,0_ corresponds to the band gap at 0 K.

The ACS is equal [9–10] to the absorption coefficient α normalized by the volume concentration of NCs, *c_V_*, (σ = α/*c_V_*). Finally, by substituting Equation 23 into the approximation presented by Kovalev et al. [29], the temperature dependence of the ACS at a fixed energy of absorbed photons, 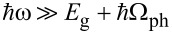, can be estimated as:

[24]
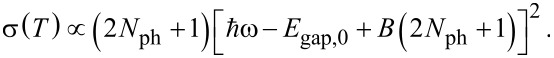


The first term on the right-hand side of Equation 24 is governed by the occupation number of phonons while the second term represents a function of the difference between photon energy and the band gap. It is also clear from Equation 24 that the higher the energy of a photon, 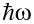, the larger the expected ACS, which is in agreement with experimental observations [14,17,25]. This can be understood by considering an increase of DOS with energy.

**Figure 5:**
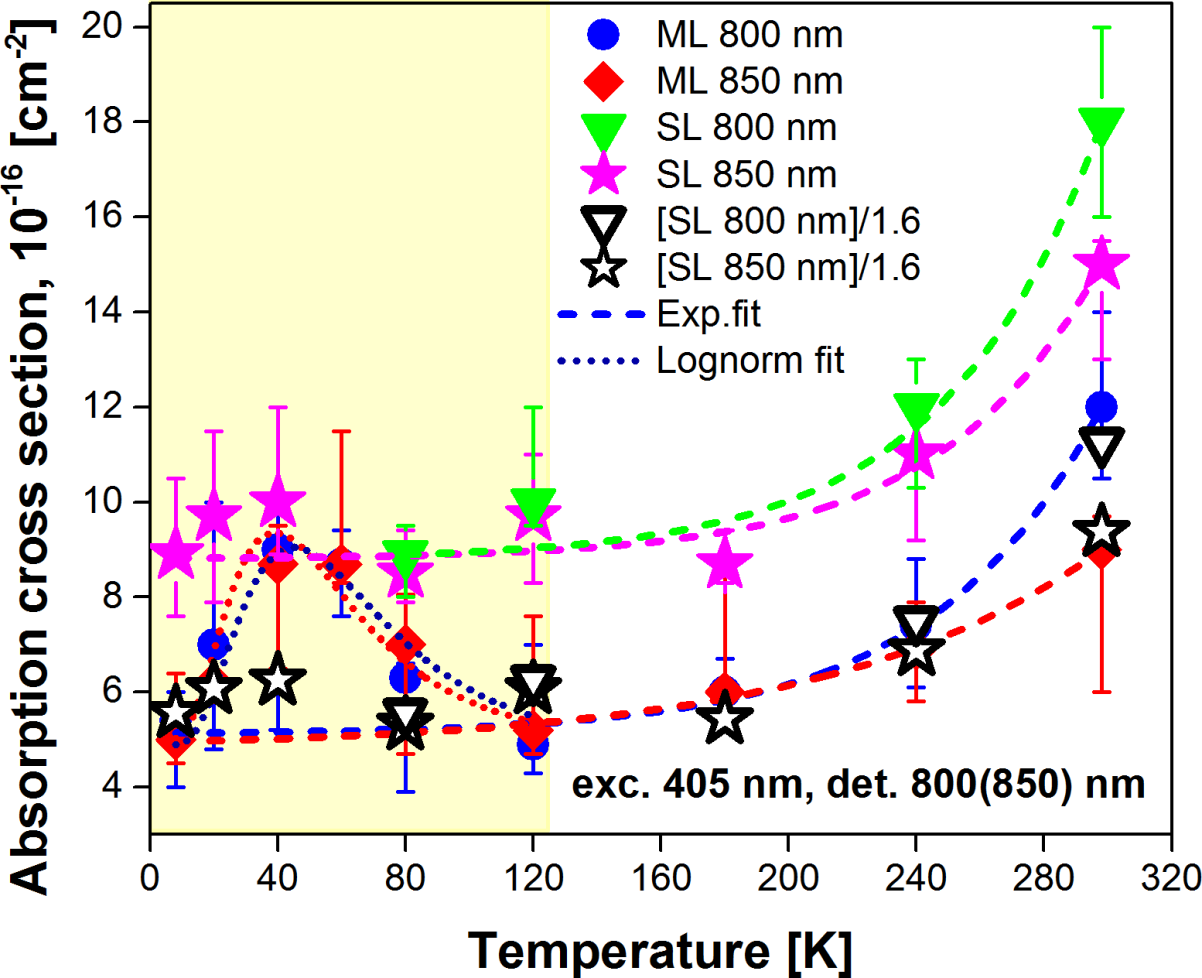
The variation of the ACS σ(*T*) of ML sample with 2.8 nm barrier thickness (circles and diamonds) and SL sample (filled triangles and stars) calculated at 800 nm (circles and upper triangles) and 850 nm (diamonds and stars). The open triangles and stars stand for the ACS of the SL sample normalized by a factor of 1.6. The dashed and dotted lines represent exponential and log-normal fits, respectively.

One of the main advantages of the PL modulation technique is that it can be relatively easily carried out at low temperatures. We experimentally measured the onset and decay curves at various sample temperatures (Figure 1b) and calculated the ACS σ(*T*) according to the procedure that was described in the previous section (Figure 5).

Besides a τ_r_-dominated temperature interval (*T* < 70 K) [21], we observed a shortening of PL lifetimes (Figure 1b) as the temperature increases due to thermally activated non-radiative processes, τ_nr_. We compared the SL sample and the ML sample with 2.8 nm barrier thickness. Recently [5], we showed by QY analysis that the SL sample consisting of a single thick layer of Si NCs (containing randomly distributed NCs) contains poorly separated NCs (comparable to the ML structure with barriers of 1 nm or thinner) in contrast to the ML sample with a thick barrier (2.8 nm). The routine was carried out with both samples at two emission wavelengths (800 and 850 nm) to obtain better statistics and avoid any experimental artefacts. Therefore, the ACS of the SL sample is expected to be comparable with the values of ML samples with separation barriers of 1 and 1.6 nm.

In contrast to Equation 24, the temperature dependence of ACS of both samples occurred to be well described by a simple exponential function in a broad interval of temperatures (Figure 5):

[25]
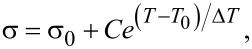


where σ_0_, *T*_0_, *C* and Δ*T* are vertical offset and horizontal shift, amplitude and characteristic temperature, respectively.

It is clear from Equation 25 and Figure 5 that the ACS σ(*T*) cannot be approximated with a constant value for all temperatures. Thus, there is decrease of the ACS to about one half when the temperature is decreased from room temperature to 120 K and the emission at 800 nm is measured. Independently of the inter-NC distance, we obtained a slight decrease of the ACS for longer emission wavelengths at room temperature (Figure 5). This result is qualitatively in agreement with the ACS trend reported by Garcia et al. and Garrido et al. [25,31] though an opposite behavior was presented in other papers [10,14] for the mentioned wavelengths. By definition, the ACS is an absorption characteristic of the excitation wavelength and should not be dependent on the emission wavelength of a NC. However, there is always a size distribution (inhomogeneous broadening) in an ensemble of NCs that results in ACS dispersion [2,10]. Assuming that NCs with a certain size emit photons at λ_em_, the ACS σ(λ_exc_, λ_em_) should be considered as a function of both excitation and emission wavelengths [14]. Notably, when decreasing the temperature of samples below ca. 150–180 K, the ACS becomes independent (Figure 5) on the emission wavelength (i.e., the NC size). Interestingly, wavelength-independent ACS values were reported by Priolo et al. even for room-temperature measurements [17]. One may notice in Figure 5 a transition from an exponential (Equation 25) to a log-normal [32] dependence of σ(*T*) for the ML sample at low temperatures. This feature must be verified in future experiments. For temperatures *T* ≥ 120 K, the ACS of the SL sample in comparison with the ML sample is ca. 1.6-times larger (Figure 5). Assuming that this difference is caused by energy transfer processes between NCs in the SL sample (while it is reduced in ML sample) we can conclude that this process should be temperature-independent at least for high temperatures (*T* ≥ 120 K).

## Conclusion

In summary, we presented a thorough study of ACS changes with inter-NC distance, σ(*x*), and temperature, σ(*T*), in Si NCs. The classical system of kinetic equations was strictly solved and the most general solution was obtained. It helped us to define the limits of the original model and correctly implement the PL modulation technique employed for ACS determination. We demonstrated that doubling the barrier thickness from about 1 to 2.2 nm results in decrease of the ACS by factor of ca. 1.5. An optimum barrier thickness of ca. 1.6 nm was calculated to obtain maximal PL brightness, which can be helpful for the construction of efficient luminescent devices. ACS changes with the barrier thickness can be due to modification of defect states and/or varying probabilities of energy transfer between NC layers. Cooling the sample below 150–180 K makes the ACS independent on the emission wavelength (800 and 850 nm). An exponential decrease of the ACS in both SL and ML samples was revealed after decreasing the temperature down to 120 K. A smaller occupation number of phonons and an expansion of the band gap of Si NCs at low temperatures were proposed to cause these phenomena.
